# Lactoferrin/Calcium Phosphate-Modified Porous Ti by Biomimetic Mineralization: Effective Infection Prevention and Excellent Osteoinduction

**DOI:** 10.3390/ma14040992

**Published:** 2021-02-19

**Authors:** Song Chen, Yuanli He, Linna Zhong, Wenjia Xie, Yiyuan Xue, Jian Wang

**Affiliations:** State Key Laboratory of Oral Diseases, National Clinical Research Center for Oral Diseases, West China Hospital of Stomatology, Sichuan University, Chengdu 610041, China; chensong789654@outlook.com (S.C.); 18328575914@163.com (Y.H.); lollipop0_0@163.com (L.Z.); Beemoxwj@outlook.com (W.X.); yiyuanxue0714@163.com (Y.X.)

**Keywords:** lactoferrin, calcium phosphate, biomimetic mineralization, titanium implants, surface modification

## Abstract

The surface modification of titanium (Ti) can enhance the osseointegration and antibacterial properties of implants. In this study, we modified porous Ti discs with calcium phosphate (CaP) and different concentrations of Lactoferrin (LF) by biomimetic mineralization and examined their antibacterial effects and osteogenic bioactivity. Firstly, scanning electron microscopy (SEM), the fluorescent tracing method, X-ray photoelectron spectroscopy (XPS), Fourier transform infrared spectroscopy (FTIR), energy dispersive X-ray spectroscopy (EDX), X-ray diffraction (XRD), and the releasing kinetics of LF were utilized to characterize the modified Ti surface. Then, the antibacterial properties against *S. sanguis* and *S. aureus* were investigated. Finally, in vitro cytological examination was performed, including evaluations of cell adhesion, cell differentiation, extracellular matrix mineralization, and cytotoxicity. The results showed that the porous Ti discs were successfully modified with CaP and LF, and that the LF-M group (200 μg/mL LF in simulated body fluid) could mildly release LF under control. Further, the LF-M group could effectively inhibit the adhesion and proliferation of *S. sanguis* and *S. aureus* and enhance the osteogenic differentiation in vitro with a good biocompatibility. Consequently, LF-M-modified Ti may have potential applications in the field of dental implants to promote osseointegration and prevent the occurrence of peri-implantitis.

## 1. Introduction

As prosthodontics and oral implantology have developed rapidly in the last few decades, indications for implant surgery have grown and dental implants have become the preferred treatment for the replacement of lost teeth. Good osteointegration [1] is a guarantee of the satisfactory prognosis of implant surgery, which is a complicated process and is easily affected by osteoblast adhesion, osteogenic differentiation, and local bacterial infection [2]. With excellent biocompatibility, mechanical properties, and corrosion resistance, titanium (Ti) has been commonly used for dental implants and bone surgery [3,4,5]. However, because of its insufficient osteoinduction and antibacterial properties [6], there is a risk of substandard osteointegration leading to dental implant failures when the condition of operative regions is poor [7]. In order to enhance the biological activities of implants to promote antimicrobial properties, pre-osteoblast adhesion, and differentiation, various surface modifications of implants have been attempted [8,9]. For instance, Wang et al. fabricated composite HA/Ag/CS nanocoatings, and three active ingredients successfully inhibited bacterial growth and promoted bone regeneration and revascularization [10].

Multiple ingredients may provide multiple effects, but they often lead to increased toxicity [11]. For instance, Xie et al. fabricated a graphene oxide/Ag/collagen composite coating and found strong cytotoxicity in a short time [12]. Moreover, when we investigated a coating fabricated from multiple components, it was usually difficult to assess and control its toxicity due to the complicated interactions of bioactive components. In order to overcome this problem, multifunctional biochemicals could be a promising solution in the field of bioengineering. Lactoferrin (LF) is a kind of glycoprotein that is widely distributed in mammals; there is about 1.13 g/L of it in human tears [13] and more than 5 g/L in human colostrums [14]. Since its discovery in the 20th century, LF and its derivatives have shown excellent antibacterial ability, so it has been widely used as an antibacterial molecule to improve the antibacterial properties of bioengineering materials. In Singh’s study, LF could prevent bacterial biofilm development by sequestering free iron [15]. In recent years, LF was reported for its potential to promote ion absorption, induce bone formation, inhibit carcinoma [16], and regulate the immune system. Therefore, as a kind of excellent multifunctional biomolecule, LF shows enormous potential for the surface modification of implants.

Although LF could influence the behaviors of osteoblasts and microbes, its stable structure is a prerequisite to fully function in implants. LF is liable to denature due to some environment factors, such as temperature, pH, and the presence of other proteins and polysaccharides [17,18,19]. Furthermore, high concentrations of LF have been reported to be cytotoxic [20]. There is a need for solutions to preserve the bioactivity of LF after binding it to a Ti surface and help it release slowly to maintain an appropriate concentration. Calcium phosphate (CaP) provides a potential means to solve these problems. Because of its chemical and biological similarities [21] with the mineral components in human hard tissues, several kinds of CaPs, such as hydroxyapatite (HA), β-tricalcium phosphate (β-TCP), and amorphous calcium phosphate (ACP), have been widely used in bone tissue engineering as an envelope biomaterial with an excellent biocompatibility [22,23,24,25]. For example, Narbat et al. successfully controlled the release of antimicrobial peptides and effectively protected their antibacterial properties [26]. Meanwhile, CaP could be fabricated at mild pH and temperature by biomimetic mineralization [27]. With the above advantages, CaP could be a useful material to protect and release LF in a controlled way.

In this study, we fabricated LF/CaP coatings on porous Ti modified with different concentrations of LF by biomimetic mineralization with the goal of finding the best solution for both antibacterial and osteoinductive properties. We characterized the LF/CaP-modified Ti discs and investigated the release kinetics of LF. Then, *S. sanguis* and *S. aureus* were incubated to assess the antibacterial ability. Meanwhile, rat bone marrow stromal cells (BMSCs) were cultured on Ti surfaces for cytological examination to investigate their effectiveness and safety for potential applications in the field of dental implants.

## 2. Materials and Methods

### 2.1. Material Preparation and Characterization

#### 2.1.1. Preparation of Alkali- and Heat-Treated Porous Ti

Commercially available pure Ti discs (10 × 10 × 1 mm^2^, >99.9%) were sequentially polished with #400, #1200, and #2000 silicon carbide papers and ultrasonically cleaned with ethylalcohol and deionized (DI) water three times. Then, the discs were etched in 4 wt.% hydrofluoric acid for 2 min and cleaned with DI water to completely remove impurities. After cleaning, the discs were subjected to alkali treatment at 60 °C for 24 h, as was reported by Wang et al. [28]. After the Ti discs cooled naturally to room temperature (about 25 °C), they were rinsed with DI water three times and dried in a vacuum drying oven at 37 °C for another 24 h. Finally, the as-prepared discs were heated in a furnace at 600 °C for 1 h.

#### 2.1.2. Pre-Calcification and LF/CaP Fabrication

A total of 0.1 mol/L CaCl_2_ solution (11.1 g anhydrous calcium chloride and 1000 mL DI water), 0.1 mol/L K_2_HP0_4_ solution (17.4 g dipotassium phosphate and 1000 mL DI water), and simulated body fluid (SBF, 8.04 g sodium chloride, 0.442 g dipotassium trihydrate phosphate, 0.438 g anhydrous calcium chloride, 1000 mL DI water, a small amount of hydrochloric acid and Tris base used to adjust pH to 7.4) were prepared in advance. Different concentrations of LF/SBFs (20, 200, and 2000 μg/mL LF) were freshly prepared. Alkali- and heat-treated porous Ti discs were firstly put into CaCl_2_ solution and then equal volumes of K_2_HP0_4_ solution were slowly dropped into it while stirring. Next, the mixed solution was stirred gently for another 15 min and then left to stand for 1 h. Subsequently, the Ti discs were rinsed with DI water 3 times to remove suspended particles. Finally, the Ti discs were placed in SBF and LF/SBFs at 37 °C for 7 days, and every 2 days the SBFs were changed. The obtained samples were lyophilized and denoted as CaP, LF-L(20 μg/mL), LF-M(200 μg/mL), and LF-H(2000 μg/mL).

#### 2.1.3. Characterization of Samples

The surface morphology of each sample (CaP, LF-L, LF-M, and LF-H) was characterized by scanning electron microscopy (SEM, KYKY Technology Development LTD, Beijing, China). Fluorescein isothiocyanate-labeled lactoferrin (FITC-LF, Ruixibio, Xi’an, China) was utilized to explore the distribution of LF on the surface of the LF/CaP coatings. The element composition and phase composition of each sample was determined by X-ray photoelectron spectroscopy (XPS, Kratos, Manchester, UK), energy dispersive X-ray spectroscopy (EDX, KYKY Technology Development LTD, Beijing, China), Fourier transform infrared spectroscopy (FTIR, Thermo Fisher Scientific, Waltham, MA, USA) and X-ray diffraction (XRD, Kratos, Manchester, UK). Additionally, we observed the surface of LF-M dried by vacuum oven at 37 °C by SEM in order to ensure that the process of lyophilization would not influence the surface morphology of the LF/CaP coatings.

#### 2.1.4. In Vitro Release Kinetics of LF

The in vitro release kinetics of LF from the LF/CaP samples were determined by the Pierce BCA Protein Assay Kit (Thermo Fisher Scientific, Waltham, MA, USA). The samples were immersed in 1 mL of phosphate buffered saline (PBS) for 6 h, 12 h, 1 day, 2 days, and 4 days at 37 °C without agitation. A total of 60 μL lixiviums was collected to examine the concentration of released LF and another 60 μL of fresh PBS was added at each time point. Finally, the lixivium at 1 month was collected and detected as the cumulative concentration of LF in each group. Additionally, we detected the concentration of each group by inductively coupled plasma-mass spectrometry (ICP-MS) at the 24th hour and 96th hour.

### 2.2. Antibacterial Evaluation

#### 2.2.1. Bacterial Strains and Culture Conditions

The antibacterial bioactivity of coatings was evaluated by streptococcus sanguis (*S. sanguis*, State Key Laboratory of Oral Diseases, Sichuan University, Sichuan, China) and Staphylococcus aureus (*S. aureus*, State Key Laboratory of Oral Diseases, Sichuan University, Chengdu, China). *S. sanguis* was cultured with brain heart infusion (BHI) culture medium (BD, New Jersey, USA) at 37 °C under anaerobic conditions, and *S. aureus* was cultured with Luria–Bertani (LB) broth medium at 37 °C under aerobic conditions.

#### 2.2.2. Bacterial Adhesion

For the adhesion experiment, *S. sanguis* and *S. aureus* were diluted with culture medium until the concentrations reached 1 × 10^7^ and 1 × 10^6^ CFU/mL [29,30], respectively, and 1 mL of bacterial culture suspension for each bacterial strain was added to the Ti specimens (CaP, LF-L, LF-M, and LF-H) in a 24-pore plate. After incubation for 4 h, the Ti discs were gently washed with PBS to remove non-adherent bacteria, and the attached bacteria were then harvested in 1 mL of medium by sonication for 5 min. A total of 10 μL of the harvested bacterial suspension from each pore were dropped onto a solid agar plate (BHI for *S. sanguis* and LB for *S. aureus*) in an orderly manner and incubated for another 24 h. The bacterial colonies were imaged by ChemiDoc MP Imaging System (Bio-Rad, Irvine, CA, USA) and counted (n = 3) in the images. To further investigate the morphology of the attached bacteria, the fixed bacteria on the Ti specimens were fixed by 2.5% glutaraldehyde, dehydrated in a graded ethanol solution (30, 50, 70, 80, 90, and 100%) for 15 min, and then observed by SEM. The experiments were conducted in triplicate.

#### 2.2.3. Bacterial Proliferation

The optimal density (OD) method [31] and spread plate method were applied to evaluate the ability to inhibit bacteria growth of Ti specimens. Specifically, 1 mL of bacteria suspension of *S. sanguis* (1 × 10^7^ CFU/mL) and *S. aureus* (1 × 10^6^ CFU/mL) was co-incubated with the specimens. After incubation for 1, 4, and 24 h, the bacteria suspension of each well was shaken for 10 min and collected. Then, the absorbance of the bacteria suspension in each pore was examined on a microplate reader (Thermo Fisher Scientific, Waltham, MA, USA) at a wavelength of 600 nm. Meanwhile, the bacteria suspensions of 4 and 24 h were diluted 10^4^ times with PBS and 100 μL of the dilution was spread onto an agar plate. After incubation for 24 h, the bacterial colonies on plates were imaged by the ChemiDoc MP Imaging System (Thermo Fisher Scientific, Waltham, MA, USA). The experiments were conducted in triplicate.

### 2.3. In Vitro Cellular Assessment

#### 2.3.1. Cell Culture

Bone marrow stromal cells (BMSCs) obtained from the femurs of 2-week-old male Sprague-Dawley (SD) rats (Animal Research Center, Sichuan University, Sichuan, China) were cultured in alpha-modified Eagle’s medium (a-MEM, Gibco, Gaithersburg, MD) containing 10% fetal bovine serum (FBS, Gibco) and 1% penicillin/streptomycin (PS, HyClone, Logan, UT, USA) at 37 °C in a 5% CO_2_ atmosphere. All the animal procedures were approved by the Animal Ethics Committee of State Key Laboratory of Oral Diseases (SCHSIRB-D-2021-004). The culture media were changed every two days and the cells were subcultured when they reached a confluence of 80–90%.

#### 2.3.2. Cell Adhesion

The BMSCs were seeded on the Ti discs to observe the cell adhesion via SEM and inverted fluorescence microscope (IFM, Leica, Weztlar, Germany). With a cell density of 8 × 10^4^ cells/mL, BMSCs were cultured on different samples (CaP, LF-L, LF-M, and LF-H) in α-MEM for 1 and 4 h. Then, the specimens were rinsed mildly with PBS for two times to remove unattached cells and separately fixed in 4% paraformaldehyde (PFA, HyClone, Logan, UT, USA) and 2.5% glutaraldehyde at RT overnight. After that, the Ti discs soaked in paraformaldehyde were permeabilized with 0.5% Trion X-100 for another 5 min at RT and then washed with PBS twice. Subsequently, the BMSCs on the Ti surface were stained with fluorescein isothiocyanate-phalloidin (rhodamine-phalloidin, Yeasen, Shanghai, China) and 4’,6-diamidino-2-phenylindole (DAPI, Beyotime, Shanghai, China) in the dark following the manufacturer’s instructions. The stained F-actin and cell nuclei were observed and imaged by IFM. On the other hand, the samples fixed in glutaraldehyde were dehydrated in graded ethanol solutions (30, 50, 70, 80, 90, and 100%) for 15 min each. After sputtering with palladium-gold, the fixed cells were observed by SEM. In order to quantitatively assess the adhesive cell number on the samples, a cell counting kit-8 (CCK-8, Solarbio, Beijing, China) assay was applied. Briefly, after totally removing the non-adhered cells with PBS, 300 mL of new culture medium with 30 mL of CCK-8 solution was added to immerse the Ti samples. After incubating at 37 °C for 2 h, the absorbance was examined on a microplate reader at a wavelength of 450 nm. The experiments were conducted in triplicate.

#### 2.3.3. Alkaline Phosphatase Activity

The alkaline phosphatase (ALP) activity was examined to evaluate the differentiation of BMSCs. An osteogenic medium was applied, consisting of α-MEN with 10% FBS, 1% PS, 100 nM dexamethasone, 50 mg/L ascorbic acid, and 10 mM Na-b-glycerophosphate. The medium was used after the BMSCs were incubated on the samples for 24 h and exchanged every two days. BSMCs were seeded on the Ti discs (CaP, LF-L, LF-M, and LF-H) at a cell density of 2 × 10^4^ cells/mL and cultured for 5 and 7 days. Then, the ALP activity of the BMSCs was measured qualitatively and quantitatively. For qualitative investigation, the samples were fixed with 4% PFA and stained with an alkaline phosphatase color development kit (Beyotime, Shanghai, China). The cells were observed by a stereomicroscope (OLYMPUS, Shinjuku, Japan). For quantitative evaluation, the BMSCs were rinsed by PBS twice and 1% Triton X-100 was added to obtain the cell lysates. Subsequently, an alkaline phosphatase assay kit (Jiancheng, Nanjing, China) was added and the results were measured with a spectrophotometer at 520 nm. Finally, the results were normalized to the total protein content measured by a BCA protein assay kit (Beyotime, Shanghai, China). The experiments were conducted in triplicate.

#### 2.3.4. Extracellular Matrix Mineralization

Extracellular matrix (ECM) mineralization was investigated by alizarin red staining. BMSCs were seeded on Ti discs (CaP, LF-L, LF-M, and LF-H) at a cell density of 1 × 10^4^ cells/mL with osteogenic medium and cultured for 7 and 14 days. Then, the samples were thoroughly rinsed with PBS twice, followed by fixing with 4% PFA at 4 °C for 1 h. After that, the cells were stained with 40 mM of alizarin red in Tris-HCl solution (pH 4.2) for 10 min at room temperature. Next, the BMSCs were thoroughly washed with DI water three times to remove excess dye and then imaged with a stereomicroscope (OLYMPUS, Shinjuku, Japan). With regard to the quantitative assessment, the stain of ECM mineralization was dissolved by 10% cetylpyridinium chloride in PBS for 15 min at RT and the absorbance at 542 nm was measured using a microplate reader. The experiments were conducted in triplicate.

#### 2.3.5. Cell Toxicity

To quantitatively evaluate the cytotoxicity of the samples, BMSCs were quantified by the CCK-8 assay and the fluorescent staining method. Cells were seeded on the samples (CaP, LF-L, LF-M, and LF-H) with a cell density of 2 × 10^4^ cells/mL; the samples were investigated by CCK-8 assay after being cultured for 1, 3, 5, and 7 days. Briefly, BMSCs were rinsed by PBS twice and 300 mL of new culture medium with 30 mL of CCK-8 solution was added to each sample. After incubating at 37 °C for 2 h, the absorbance was examined on a microplate reader at a wavelength of 450 nm. Cellular toxicity was also evaluated by a Calcein-AM/PI double-staining kit (Solarbio, Beijing, China). After incubation for 1 or 3 days, the BMSCs on the Ti plates were stained by calcein-AM and propidium iodide following the manufacturer’s instructions and observed by IFM. Live and dead cells appeared as green and red, respectively. For the quantitative analysis, the live/dead cells of each group were counted in random fields (n = 5) with equal areas in the fluorescent images and the cell viability was calculated accordingly. The experiments were conducted in triplicate.

### 2.4. Statistical Analysis

Statistical analysis was conducted using IBM SPSS version 19.0. One-way analysis of variance (ANOVA) and the Student–Newman–Keuls test were used for multiple comparisons. Differences were considered statistically significant at *p* < 0.05.

## 3. Results

### 3.1. Surface Characterization and LF Release Profile

#### 3.1.1. Surface Characterization

**Figure 1:**
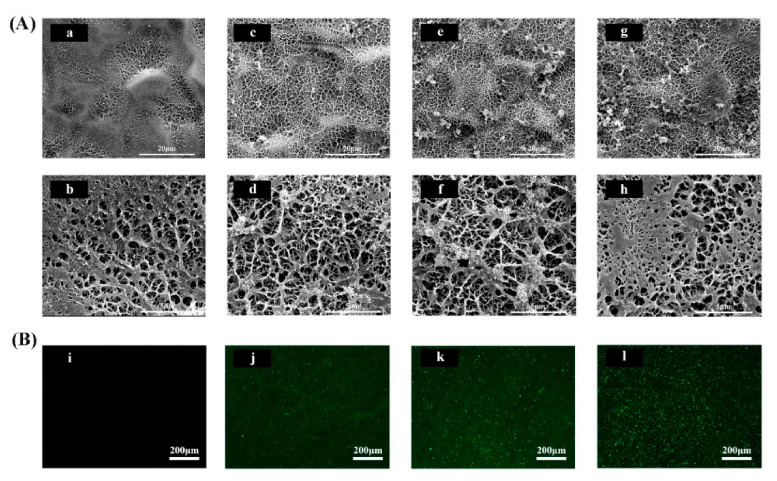
(**A**) SEM images of the calcium phosphate (CaP, **a**,**b**), lactoferrin-low(LF-L, **c**,**d**), lactoferrin- middle (LF-M, **e**,**f**), and lactoferrin-high (LF-H, **g**,**h**) surfaces; (**B**) fluorescein isothiocyanate labeled lactoferrin (FITC-LF) distribution on the CaP (**i**), LF-L (**j**), LF-M (**k**), and LF-H (**l**) surfaces.

The surface morphologies of the as-prepared Ti discs (CaP, LF-L, LF-M, and LF-H) were observed by SEM. All of the specimens exhibited a porous surface morphology. As shown in Figure 1A, uniform meshy coatings and microporous structures could be observed on the surface of the alkali- and heat-treated porous Ti discs. On the SEM images of LF-modified groups (LF-L, LF-M, and LF-H), some crystal clusters could be observed, which was different from the CaP group. With a higher concentration of LF in the SBFs, more floral crystals and denser reticular structures could be found. On the high-magnification SEM images, a similar tendency could be observed. From the fluorescence microscopy images of the Ti surface (Figure 1B), a uniform distribution of FITC-LF could be observed and more protein aggregations could be found with the increase in FTIC-LF concentration. Additionally, the SEM images (Figure S1) of LF-M dried by a vacuum oven were similar to the lyophilized LF/CaP coatings, which were microporous with some crystal clusters.

**Figure 2:**
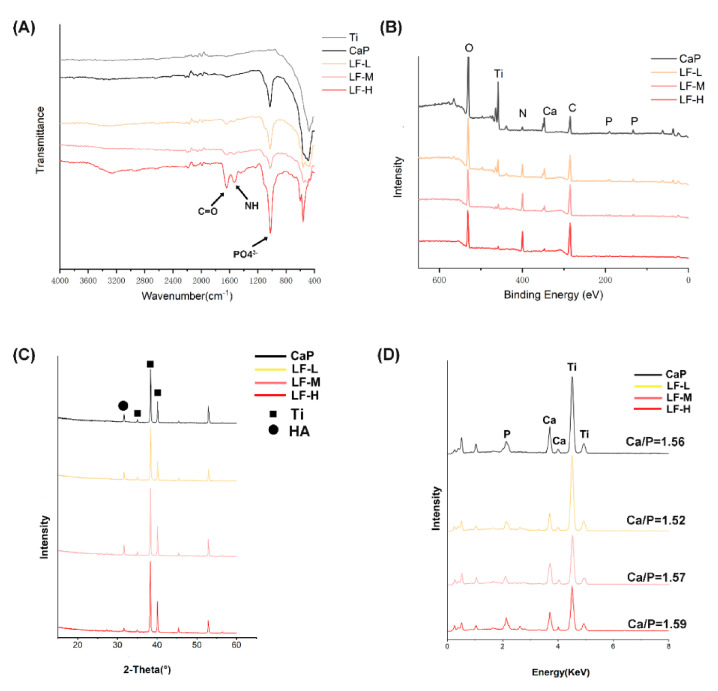
(**A**) FTIR spectra of Ti, CaP, LF-L, LF-M, and LF-H; (**B**) XPS spectra of CaP, LF-L, LF-M, and LF-H; (**C**) XRD spectra of Ti, CaP, LF-L, LF-M, and LF-H; (**D**) EDX spectra and Ca/P ratios of Ti, CaP, LF-L, LF-M, and LF-H.

To verify the successful deposition of the LF/CaP coatings and further compare the difference between samples, FTIR, XPS, XRD, and EDX were applied. In the FTIR spectra (Figure 2A), the characteristic split peaks of PO4^3−^ were observed at 1037 cm^−1^ in CaP and all LF/CaP. Meanwhile, the characteristic split peaks of 1654 and 1541 cm^−1^ were assigned to the C=O stretching vibration of –NHCO– and the N–H bending of –NH_2_ of LF in LF/CaP coatings. Additionally, only LF-H exhibited a significant peak at around 3300 cm^−1^. As the XRD spectra (Figure 2C) show, characteristic peaks of Ti and HA could be observed in all groups, but the peak of HA in LF-H was significantly weaker than in the other groups. In Figure 2B, the XPS survey scan proved the existence of Ti, O, Ca, P, C, and N peaks. With the increase in LF concentration, a stronger N peak was observed but the Ti peak values decreased. Similarly, in the EDX spectra (Figure 2D) the peaks of Ti in LF-H were not as strong as those of CaP, LF-L, and LF-M. The Ca/P ratios which were calculated according to the EDX analysis were 1.56, 1.52, 1.57, and 1.49, respectively.

#### 3.1.2. In Vitro Release Kinetics of LF

**Figure 3:**
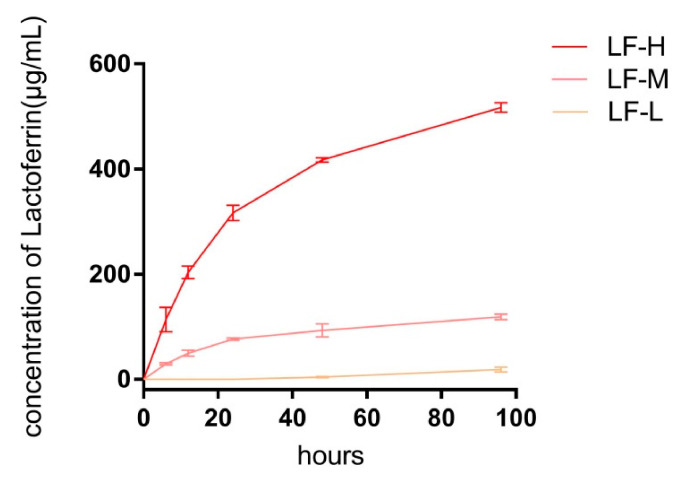
Release kinetics of lactoferrin (LF) on Ti specimens (LF-L, LF-M, and LF-H) in phosphate buffer saline (PBS).

The release kinetics of LF are shown in Figure 3. The accumulative concentrations of released LF at the 4th day were 18.39, 118.64, and 516.84 μg/mL (LF-L, LF-M, and LF-H). On the first day, the accumulative concentration of LF in the LF-H group exceeded 300 μg/mL with an initial burst release, and slowly reached up to 516.84 μg/mL in the next 3 days. With regard to the LF-L group, the released LF was not effectively detected until the 2nd day and reached 18.39 μg/mL at the 4th day. In the LF-M group, 76.46 μg/L of LF was released on the first day, which rose to 118.64 μg/mL in the following 3 days without a burst release effect. Meanwhile, as shown in Table S1 and Figure S1, the release kinetics of LF-M in percentage were also more flat than those of LF-H.

As shown in Table S2, at the 24th hour 26.2, 27.3, 25.7, and 33.3 mg/L of Ca ions were detected separately from CaP, LF-L, LF-M, and LF-H, and the concentrations of Ca ions were 39.1, 37.2, 36.9, and 39.5 mg/L after 96 hours. Additionally, the concentration ratios were 67.0%, 73.4%, 69.6%, and 84.1%, respectively.

### 3.2. Antibacterial Abilities

**Figure 4:**
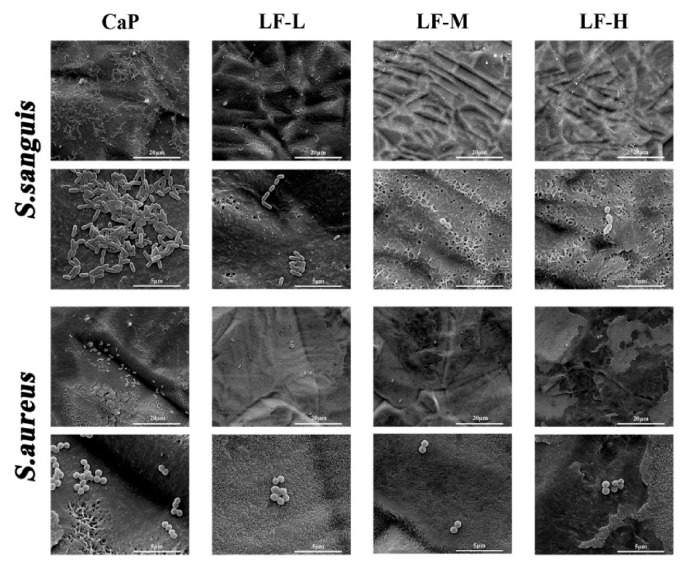
SEM images of adhesive *S. sanguis* and *S. aureus* on CaP, LF-L, LF-M, and LF-H samples.

The adhesion and proliferation of *S. sanguis* and *S. aureus* in the control group (CaP) and experimental groups (LF-L, LF-M, and LF-H) were assessed. To investigate the early adhesion of *S. sanguis* and *S. aureus*, the morphology of bacteria on the specimens was observed by SEM. As shown in Figure 4, numerous *S. sanguis* and *S. aureus* could be found on the control group; meanwhile, in the experimental groups significantly less *S. sanguis* and *S. aureus* adhered to the coatings.

**Figure 5:**
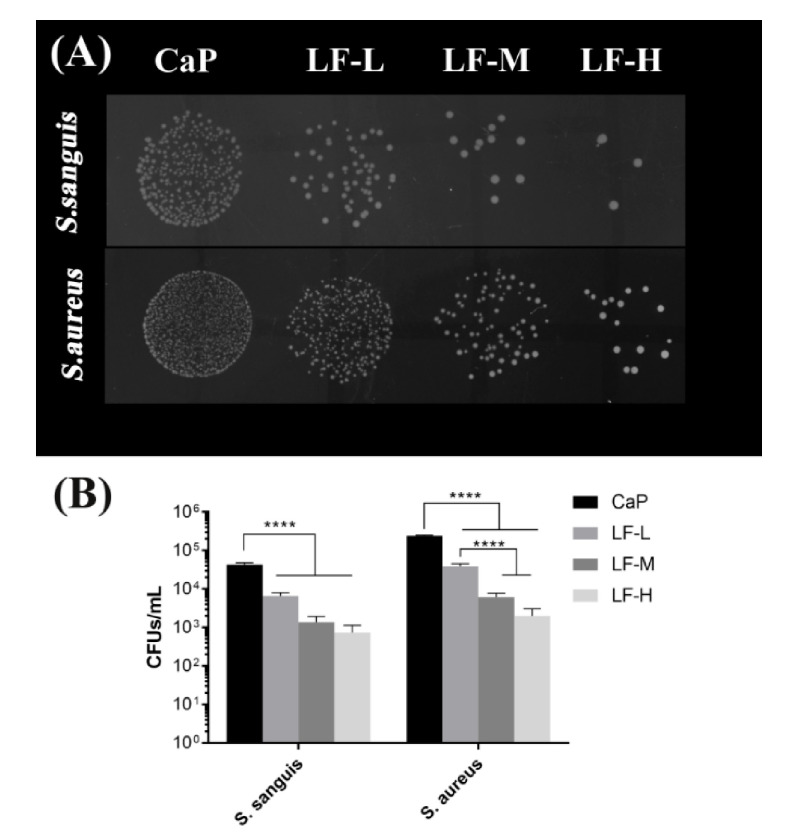
Spot assay (**A**) and colony-forming units (CFUs, **B**) of adhesive *S. sanguis* and *S. aureus* on CaP, LF-L, LF-M, and LF-H specimens cultured for 24 h. **** *p* < 0.0001.

The results of the spot assay and bacterial counting are shown in Figure 5. The *S. sanguis* colonies of the experimental groups were remarkably smaller than those of the CaP group. Compared with CaP, approximately 1Log decrease was recorded for LF-L, and a 2Log decrease to 3Log decrease was record for LF-M and LF-H. The results for *S. aureus* exhibited a similar tendency.

**Figure 6:**
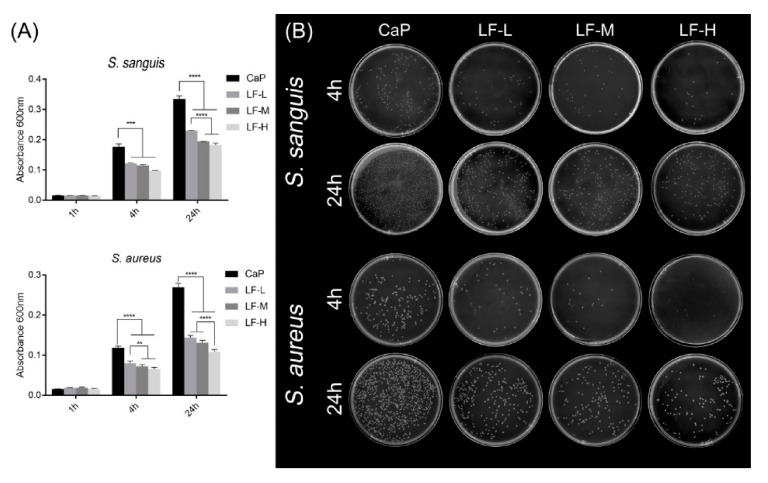
(**A**) Proliferation of *S. sanguis* and *S. aureus* cultured on CaP, LF-L, LF-M, and LF-H samples for 1, 4, and 24 h. ** *p* < 0.01; *** *p* < 0.001; **** *p* < 0.0001.(**B**) *S. sanguis* colonies and *S. aureus* colonies on the agar plates, which were incubated with CaP, LF-L, LF-M, and LF-H samples for 4 and 24 h.

The results for the antibacterial proliferation are shown in Figure 6. The absorbance of each sample was at a low level in the 1st hour (Figure 6A). After culturing for 4 and 24 h, both the results of *S. sanguis* and *S. aureus* showed that the absorbance of the CaP group was significantly higher than that of the other groups (LF-L, LF-M, and LF-H). Figure 6B shows the results of the spread plate method. The bacterial colonies (*S. sanguis* and *S. aureus*) of the experimental groups (LF-L, LF-M, and LF-H) were smaller than those of the control group (CaP), and even fewer bacterial colonies could be observed on LF-M and LF-H, no matter whether the bacteria was incubated with Ti samples for 4 or 24 h.

### 3.3. In Vitro Cell Behavior

#### 3.3.1. Cell Adhesion 

**Figure 7:**
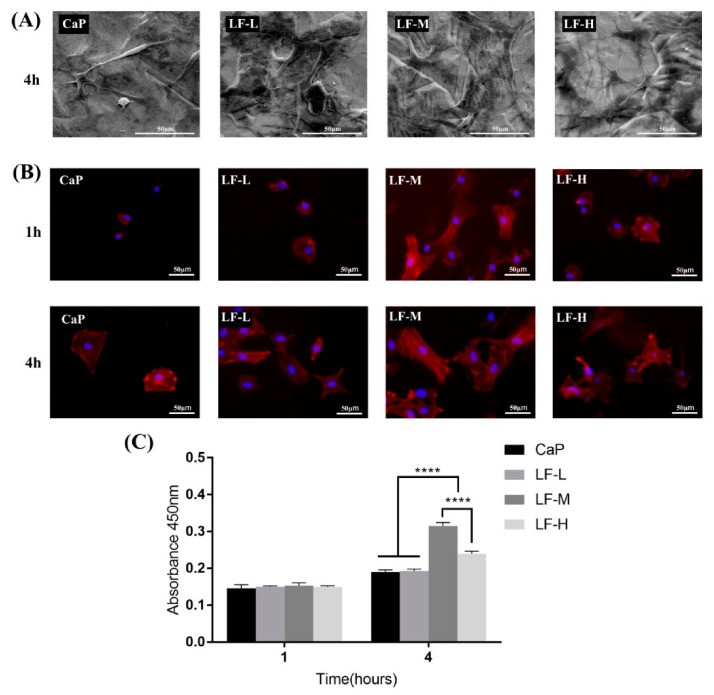
(**A**) SEM images of bone marrow stromal cells (BMSCs) cultured on CaP, LF-L, LF-M, and LF-H samples; (**B**) fluorescence microscopy images of BMSCs cultured on CaP, LF-L, LF-M, and LF-H samples for 1 and 4 h; F-actin was stained with rhodamine (red) and the nucleus with DAPI (blue); (**C**) quantitative cell early adhesion of BMSCs on CaP, LF-L, LF-M, and LF-H samples. **** *p* < 0.0001.

The morphology of the BMSCs seeded on Ti discs was observed by SEM and IFM. As Figure 7A shows, spindle-like BMSCs could be seen on the CaP group, while cells on the LF-L, LF-M, and LF-H groups exhibited a more stretched morphology with pseudopodia of filament or sheet shapes. Fluorescence microscopy images of the initial cell adhesion on the Ti specimens (CaP, LF-L, LF-M, and LF-H) are shown in Figure 7B. The BMSCs on the CaP, LF-L, and LF-H groups presented a sphere-like and polygon shape morphology with few filopodia extensions in the 1st hour; with regard to the LF-M group, polygonal cells with obvious filopodia extensions could be observed. After incubation for 4 h, stellate cells of the LF-L, LF-M, and LF-H groups were observed and the CaP group still exhibited a polygon shape. In the quantitative analysis of early adhesion, significantly more BMSCs attached to the LF-M and LF-H groups after culturing for 4 h, and the cell number of the LF-M group was even higher than that of the LF-H group (Figure 7C).

#### 3.3.2. Osteogenic-Related Assay

**Figure 8:**
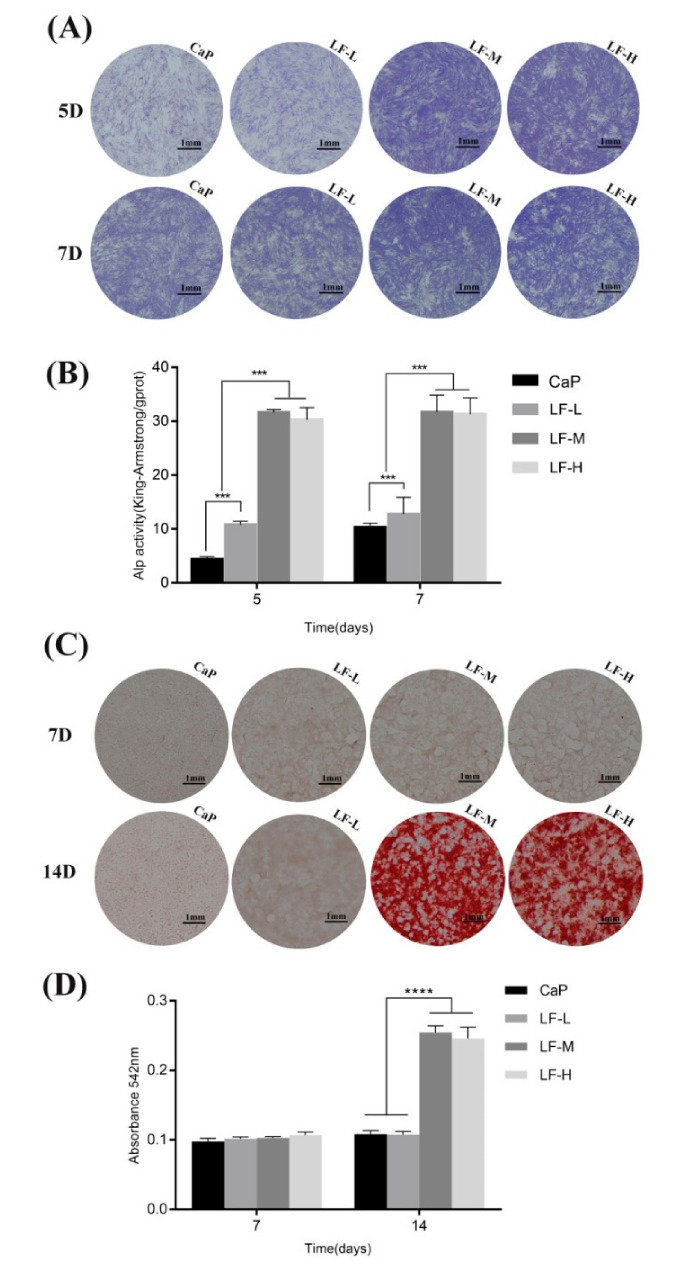
(**A**) Alkaline phosphatase (ALP) staining of BMSCs of CaP, LF-L, LF-M, and LF-H groups for 5 and 7 days; (**B**) quantitative ALP activity of the BMSCs of the CaP, LF-L, LF-M, and LF-H groups for 5 and 7 days; (**C**) matrix mineralization of BMSCs of the CaP, LF-L, LF-M, and LF-H groups for 7 and 14 days; (**D**) colorimetric quantitative results of extracellular matrix mineralization. *** *p* < 0.001, **** *p* < 0.0001.

To investigate the early differentiation of BMSCs on the coatings, qualitative ALP staining and quantitative ALP analysis was applied. The concentration of ALP (Figure 8B) in the LF-M and LF-H groups was significantly higher compared with the CaP and LF-L groups at the 5th and 7th days. Meanwhile, the ALP secretion of BMSCs from the LF-M and LF-H groups presented no significant differences. The results of the ALP staining revealed a similar tendency (Figure 8A). As for the ECM mineralization of BMSCs, there seemed to be no difference among the coatings at day 7. However, after incubation for 14 days the LF-M and LF-H groups exhibited significantly more stained calcifying nodules (Figure 8C,D).

#### 3.3.3. Cell Toxicity

**Figure 9:**
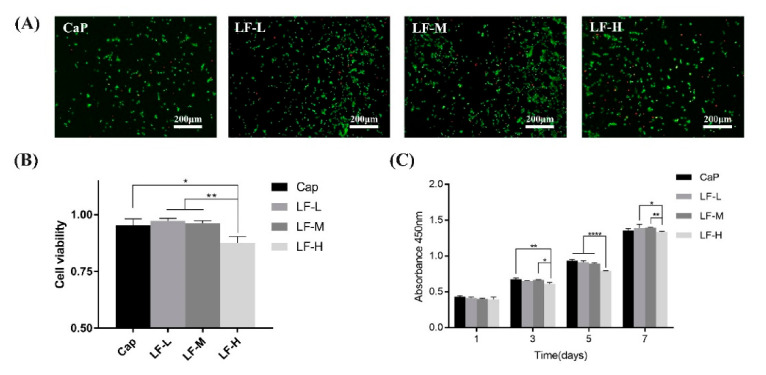
(**A**) Fluorescence microscopy images of live/dead cells (green: live, red: dead) of BMSCs after seeding on CaP, LF-L, LF-M, and LF-H for 3 days; (**B**) cell viability quantitative analysis according to the live/dead cell numbers; (**C**) the absorbance of live cells after 1, 3, 5, and 7 days of culture using the CCK-8 assay kit; * *p* < 0.05, ** *p* < 0.01, **** *p* < 0.0001.

The viability and proliferation of the BMSCs cultured on the samples (CaP, LF-L, LF-M, and LF-H) were evaluated by live/dead assay kit and cell counting kit-8 (CCK-8) analysis. The live/dead cell result in Figure 9A shows that the BMSCs were viable on all samples from the fluorescence microscopy images after 3 days of culture. The statistical analysis of the cell viability on the Ti specimens in Figure 9B indicates that all the samples exhibited a similar cell viability, except for LF-H, which was about 8% lower than CaP. Additionally, the proliferation of BMSCs on the Ti discs evaluated by a CCK-8 assay kit (Figure 9C) shows a similar trend in the results of the live/dead assay. On the first day, the cells grew well on all samples; after incubation for 3 and 5 days, the proliferation of cells of LF-H was significantly less than that of CaP and LF-M; at the 7th day, the proliferation rate of LF-H was still lower than that of LF-L and LF-M.

## 4. Discussion

In this study, we successfully modified porous Ti discs with CaP and different concentrations of LF by biomimetic mineralization and compared the LF release kinetics and antibacterial and osteoinductive ability. LF is apt to lose its vitality under the condition of strong acid/base or high temperature [32], and a high concentration of LF has a certain cytotoxicity [20]. Biomimetic mineralization may provide a simple and effective way to solve the above problems [27]. Before being modified with LF/CaP, Ti discs were subjected to standard acid-etched/alkali heat treatment to obtain a porous Ti surface and titanate layer, which was beneficial for the deposition of calcium ions by providing sufficient mechanical and chemical bonding [33]. Additionally, the pre-calcification treatment could accelerate the process of CaP mineralization and contribute to the faster formation of uniform LF/CaP coatings [34]. 

In the SEM images of the Ti surface (Figure 1A), different surface morphologies could be observed, which might have been caused by different LF concentrations in SBFs. Pan et al. found that some functional groups, such as–OH, –NH_2_, and–COO^−^, could interact with CaP crystallite during the process of biomimetic mineralization [35], and protein might provide extra crystal nuclei and form a CaP-packaged protein structure. In the process of biomimetic mineralization, a crystal nucleus were firstly formed on the porous Ti and then turned into reticular and porous structures [36,37], as exhibited in Figure 1A and Figure S1. The structure is exactly what we expected to protect the bioactivity of LF and control its release kinetics. From the fluorescence microscopy images of LF/CaP surfaces (Figure 1B), more large FITC-labeled particles could be observed in LF-H, which might have been caused by LF aggregation. Thus, different concentrations of LF might influence the crystallization process of CaP. To further determine which kind of CaP was formed, FTIR, XPS, XRD, and EDX were applied. According to the FTIR and XRD results (Figure 2A,C), HA coatings were successfully mineralized on porous Ti discs. In the FTIR spectra of LF-H, a peak around 3300 cm^−1^ was found, which was different from CaP, LF-L, and LF-M. In related studies [38], nanocrystalline HA particles exhibited broad bands around 3200–3500 cm^−1^, but crystalline HA particles did not. Accordingly, we speculated that superfluous LF in LF-H provided plentiful crystallization centers of HA and resulted in a LF/HA coating consisting of LF-HA nano-particles. The other groups were LF/HA coatings consisting of crystalline HA particles. Likewise, the weaker characteristic peak of HA in the XRD spectra of XRD indicated that a large amount of LF will lead to the poor crystallization of HA. In the results of XPS and EDX, with the increase in the LF concentration, less Ti, titanium oxide, and CaP was exposed and more LF was detected on the surface of Ti. Interestingly, in the XPS spectra of the control group (CaP), a weak N peak could be observed, which might presumably have been caused by the retention of a Tris base for regulating pH in the SBF solution. The standard Ca/P ratio of HA was 1.67 and the lower surface Ca/P ratios of our coatings may be a result of surface disorder [39]. Figure 3 presents the cumulative concentration of LF and its release kinetics, which confirmed that different LF concentrations in SBF could lead to different release kinetics of LF from the experimental groups. Superfluous LF (LF-H) in SBF may result in its explosive release. Meanwhile, the result for ICP (Table S1) also indicated the fast release of Ca ions in LF-H, which may be related to the degree of mineralization.

On one hand, we expected that LF could release slowly to avoid potential complications, such as cytotoxicity caused by local high concentrations of LF. On the other hand, sufficient LF was necessary for antibacterial and osteoinductive effects. Accordingly, we tried to find a balance point between bioactivities and biosafety by in vitro antibacterial and cellular tests.

On the basis of Figure 4, Figure 5 and Figure 6, all the experimental groups (LF-L, LF-M, and LF-H) exhibited an excellent ability to inhibit the adhesion of *S. sanguis* and *S. aureus*, and the LF-M and LF-H groups performed even better. Meanwhile, the growth of *S. sanguis* and *S. aureus* was also significantly inhibited with the rise in the LF concentration in Ti samples. Based on the above results, we could determine that LF on the Ti discs still remained bioactive as expected, in which the LF-M and LF-H groups performed better than LF-L. The formation of dental plaque is the primary step in periodontitis and peri-implantitis, and the first colonizers on implant surfaces are streptococci [40]. In another study of Persson and Renvert [41], a cluster of bacteria from implant surfaces was found to be associated with peri-implantitis, especially *S. sanguis* and *S. aureus*. Therefore, the inhibitory effects on *S. sanguis* and *S. aureus* played an important role in inhibiting the colonization of oral bacteria and decreasing the risk of peri-implantitis. 

As a kind of initial cell behavior on the implant–bone interface, early adhesion plays an important role in BMSCs’ proliferation and differentiation [42]. Thus, researchers have tried to improve cell adhesion in various ways, such as through topological structure design and surface chemistry design [43]. According to Figure 7, the early adhesion of BMSCs could be influenced by LF on Ti samples. Proper LF concentration (LF-M) promoted this process qualitatively and quantitatively, but superfluous LF (LF-H) performed even worse than LF-M, which might have been caused by its biotoxicity at high concentrations. Therefore, LF on Ti was beneficial to the early proliferation and differentiation of pre-osteoblasts. As the ALP activity showed, the BMSCs of LF-M and LF-H groups secreted a significantly higher level of ALP at day 5 and day 7. Interestingly, both the qualitative and quantitative results indicated that the ALP activity of the control group (CaP) obviously increased from day 5 to day 7. However, LF-M and LF-H did not exhibit a similar trend, and their ALP activity already reached a relatively high level at the 5th day. The abundant early secretion of ALP could be considered as the symbol that LF effectively promoted BMSCs’ early osteogenic differentiation. Additionally, the investigation of extracellular matrix mineralization also reminded us that using a proper concentration of LF could influence the later stage of osteogenic differentiation. In a word, the cell differentiation and cell mineralization of LF-M and LF-H groups were significantly enhanced compared with CaP and LF-L groups.

Although the LF-M and LF-H groups could both effectively inhibit bacterial infection and promote osteogenic differentiation, the results of the CCK-8 assay still reminded us of the potential cytotoxicity of LF. In the research of Xu et al. [20], 25 μΜ (around 2.2 mg/mL) of LF could significantly lead to the apoptosis of SGC-7901 human stomach cancer cells. As Figure 9 shown, the viability and proliferation of BMSCs of the LF-H group were slightly inhibited, which indicated its certain cytotoxicity. From the results of the LF release kinetics, we found that LF was rapidly released from the LF-H coating at the early stage, which might lead to the growth inhibition effect of BMSCs. The concentration of LF gradually decreased with the lapse of time, and its cytotoxicity concomitantly declined. In conclusion, the LF-M group presented excellent antibacterial and osteoinductive abilities and could maintain balance between bioactivity and safety.

## 5. Conclusions

In summary, we successfully modified porous Ti discs with CaP and different concentrations of LF by biomimetic mineralization and compared their LF release kinetics and antibacterial and osteoinductive ability. Compared with the CaP, LF-L, and LF-H groups, the LF-M group not only effectively inhibited the early adhesion and proliferation of *S. sanguis* and *S. aureus* but also presented a better ability to improve the early adhesion and differentiation of BMSCs in vitro with a relatively slow LF release. These results suggested that biomimetic mineralization could provide a simple and effective way to modify Ti implants with LF and protect the protein activity; a proper concentration of LF (LF-M)-modified Ti may be applied in the field of dental implants to promote osseointegration and prevent the occurrence of peri-implantitis.

## Data Availability

Data is contained within the article.

## Supplementary Materials

The following are available online at https://www.mdpi.com/1996-1944/14/4/992/s1: Figure S1: SEM images of LF-M dried by vacuum oven at 37 °C; Table S1: Cumulative concentration of lactoferrin at 1 month; Figure S2: Release kinetics of LF on Ti specimens (LF-L, LF-M and LF-H) in PBS in percentage; Table S2: Ca ions released from the coatings detected by ICP-MS.
