# Supplementary material for: Lactoferrin/Calcium Phosphate-Modified Porous Ti by Biomimetic Mineralization: Effective Infection Prevention and Excellent Osteoinduction
Source: Materials (Basel). 2021 Feb 19;14(4):992. doi: 10.3390/ma14040992 (PMC7923298; doi:10.3390/ma14040992)
Supplement: Supplementary file 1 [file materials-14-00992-s001.zip › materials-1072849-supplementary.pdf]

materials

MDPI

Supplementary Materials

Lactoferrin/Calcium Phosphate-Modified Porous Ti by Biomimetic Mineralization: Effective Infection Prevention and Excellent Osteoinduction

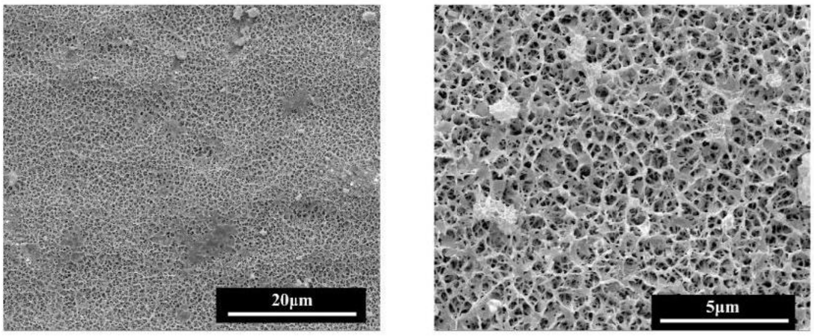

Figure S1. SEM images of lactoferrin-middle (LF-M) dried by vacuum oven at 37 °C.

Table S1. Cumulative concentration of lactoferrin at 1 month.

Group	LF-L	LF-M	LF-H
Concentration ±SD (μg/mL)	24.81 ±2.73	133.29 ±3.53	521.03 ±8.83

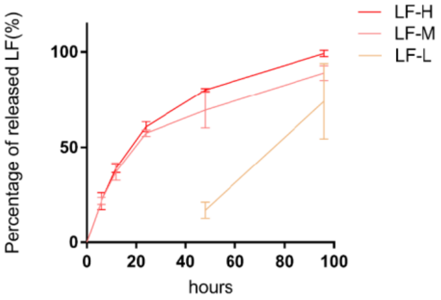

Figure S2. Release kinetics of LF on Ti specimens (LF-L, LF-M and LF-H) in PBS in percentage.

Table S2. Ca ions released from the coatings detected by inductively coupled plasma-mass spec-trometry (ICP-MS).

Group	Element	Time (h)	Concentration (mg/L)	Concentration ratio (24 h/96 h)
CaP	Ca	24	26.2	67.0%
		96	39.1	
LF-L	Ca	24	27.3	73.4%
		96	37.2	
LF-M	Ca	24	25.7	69.6%
		96	36.9	
LF-H	Ca	24	33.3	84.1%
		96	39.5	
